# Assessing the Feasibility of Music Therapy During Exercise in Cardiac Rehabilitation: A Mixed-Methods Analysis

**DOI:** 10.1093/geroni/igaf122.4070

**Published:** 2025-12-31

**Authors:** Ryan Conard, Wen-Chih Wu

**Affiliations:** The Warren Alpert Medical School of Brown University, Providence, Rhode Island, United States; Brown University Health, Providence, Rhode Island, United States

## Abstract

Cardiac rehabilitation (CR) is an evidence-based secondary prevention strategy following cardiovascular events. However, appointment adherence is an ongoing challenge, often limited by psychosocial barriers. Music therapy has shown promise in improving emotional well-being, and enhancing exercise endurance while lowering perceived exertion, which could improve experience of CR sessions. This quality improvement pilot project evaluated the feasibility of offering personalized music listening during exercise sessions as a novel and underexplored approach to increasing participation in CR. Over an eight-week period, all patients in a 12-week CR program were offered a personalized music intervention during supervised exercise three times a week. Participants could opt in or out at each session. Playlists were generated using participant preferences and offered via MP3 players or personal devices. Uptake, adherence, and qualitative feedback were tracked across sessions. 160 people participated (65.6% male, mean age 67.7±12.1 years, 80.6% white). 58.4% agreed to listen to music (uptake). Of these, 97.5% continued to adhere to the music intervention across their remaining sessions. An average of 61.5% of CR participants used music daily. Qualitatively, people reported highly enjoying the intervention, characterizing the music as energizing, motivating, and relaxing. Many reported that music motivated them to stay longer each session or return more often. The high uptake and adherence of participants, positive qualitative feedback, minimal disruption to existing clinic operations, and low cost ($21 per MP3 player) illustrate the strong feasibility and suggest that personalized music during exercise may be a scalable strategy to enhance CR engagement and warrant further study.

